# A double-blind, randomized, placebo-controlled trial of combined calcitriol and ergocalciferol versus ergocalciferol alone in chronic kidney disease with proteinuria

**DOI:** 10.1186/s12882-017-0436-6

**Published:** 2017-01-14

**Authors:** Paweena Susantitaphong, Siriwan Nakwan, Sadudee Peerapornratana, Khajohn Tiranathanagul, Pisut Katavetin, Nattachai Srisawat, Kearkiat Praditpornsilpa, Somchai Eiam-Ong

**Affiliations:** Division of Nephrology, Department of Medicine, King Chulalongkorn Memorial Hospital, Faculty of Medicine, Chulalongkorn University, 1873, Rama 4 Rd., Pathumwan, Bangkok, 10330 Thailand

**Keywords:** Vitamin D deficiency, CKD, Proteinuria, Ergocalciferol, Calcitriol

## Abstract

**Background:**

KDOQI guideline suggests that nutritional vitamin D should be supplemented in chronic kidney disease (CKD) patients who have vitamin D insufficiency/deficiency. However, there are scarce data regarding the additional benefit of active vitamin D supplement in CKD patients who were receiving nutritional vitamin D supplement. This study was conducted to explore the effect of adding active vitamin D to nutritional vitamin D supplement on proteinuria and kidney function in CKD with vitamin D insufficiency/deficiency.

**Methods:**

This double-blind, randomized placebo-controlled trial was performed to answer the above question. Sixty-eight patients with CKD stage 3–4, urine protein to creatinine ratio (UPCR) > 1 g/g, and serum 25OH-D level < 30 ng/mL were enrolled. Patients were randomly assigned to receive 12-week treatment with oral ergocalciferol plus placebo (*n* = 36) or oral ergocalciferol plus calcitriol (*n* = 32).

**Results:**

The mean baseline values of UPCR of both groups were comparable (3.6 ± 3.8 g/g in combined group and 3.5 ± 3.0 g/g in ergocalciferol group). Following 12-week treatment, there were significant reductions in UPCR from baseline in both groups (2.3 ± 2.1 g/g in combined group and 2.4 ± 2.0 g/g in ergocalciferol group). The percentage reductions in UPCR of both groups were not significantly different. The mean eGFR and blood pressure did not differ between baseline and 12-week follow-up and between both groups. No severe hypercalcemia or serious side effects were noted in both groups.

**Conclusions:**

The proteinuria lowering effect of ergocalciferol in CKD patients with vitamin D deficiency was demonstrated. Additional calcitriol supplement did not have more effects on proteinuria.

**Trial registration:**

(Thai Clinical Trials Registry (TCTR) 20140929002). Date of registration: September 27, 2014.

## Background

Chronic kidney disease (CKD) is one of the most extremely important non-communicable diseases that has significant morbidity and mortality consequences [1]. The major complications related to CKD include cardiovascular disease, infectious complications, and mineral and bone disorder (MBD). A recent meta-analysis demonstrated that lower estimated glomerular filtration rate (eGFR) and higher albuminuria were each independently associated with end-stage renal disease (ESRD) and mortality. Both eGFR and albuminuria were more strongly associated with ESRD than mortality in CKD patients [2].

Vitamin D deficiency/insufficiency is a common problem in CKD patients due to dysregulation of vitamin D metabolism from renal insufficiency [3]. Although the definite critical serum 25 (OH) D level and benefits of 25 (OH) D supplement in CKD patients remain controversial, In the 2003 Kidney Disease Outcomes Quality Initiative (KDOQI) guideline suggested that patients with serum 25(OH) D levels < 30 ng/mL should receive supplementation with nutritional vitamin D. The dosage regimens were recommended to be the same as general population [4].

Besides the classical action of vitamin D as an important regulator of mineral bone metabolism, recent evidence points to other important functions in different target organs including renal, cardiovascular systems, and immune response regulation [5]. Vitamin D deficiency was associated with a higher annual incidence of albuminuria, decreased eGFR, and independently predicted 5-year incidence of albuminuria [6]. Vitamin D deficiency was also a significant risk factor for the development of CKD stage 3–5 [7, 8]. In addition, vitamin D deficiency is independently associated with a higher risk of 50% increase in baseline serum creatinine, ESRD, or death in patients with type II diabetic nephropathy (DN) [9].

Previous cohort studies showed that daily cholecalciferol, a nutritional vitamin D, supplement had a beneficial effect in decreasing albuminuria in CKD and DN [10, 11]. Active vitamin D has been demonstrated to lessen renin-angiotensin aldosterone system (RAAS) and intraglomerular pressure, and might ameliorate renal injury by reducing fibrosis, apoptosis, and inflammation in animal models [12]. Several randomized controlled trials in patients with proteinuric kidney disease demonstrated the benefit of calcitriol or paricalcitol supplement in decreasing proteinuria [13–15]. However, there were no randomized controlled trials (RCTs) exploring the effect of the additional benefit of active vitamin D supplement in CKD patients who were receiving nutritional vitamin D supplement. Therefore, this study was conducted to explore the effect of combined nutritional vitamin D and active vitamin D supplement on proteinuria and kidney function in CKD with vitamin D insufficiency/deficiency.

## Methods

### Study design and participants

The study was performed during July 2014 and February 2015 in adult Thai CKD patients with proteinuria and vitamin D insufficiency/deficiency at the outpatient clinic at King Chulalongkorn Memorial Hospital (KCMH), Bangkok, Thailand. The study was approved by the Institutional Review Board of the Faculty of Medicine, Chulalongkorn University (Bangkok, Thailand; IRB.093/57) with clinical trial registration (Thai Clinical Trials Registry (TCTR) 20140929002; date of registration: September 27, 2014). All participants received information of study details before giving written informed consent. The inclusion criteria included eGFR of 15–60 mL/min per 1.73 m^2^, age 18 years or older, proteinuria greater than 1 g/day, and serum vitamin D (25-OH) level less than 30 ng/mL. The eGFR was calculated with serum creatinine concentrations by using Thai-MDRD formula [16]. Exclusion criteria included active glomerulonephritis, receiving immunosuppressive drugs within 3 months before enrollment, adjusting RAAS blockade such as angiotensin-converting-enzyme inhibitors or angiotensin II receptor blockers (ACEIs/ARBs) within 3 months before enrollment, and serum calcium level more than 10.2 mg/dL.

This study was a double-blind RCT of ergocalciferol, a nutritional vitamin D, with or without calcitriol, an active vitamin D, in CKD with proteinuria and vitamin D insufficiency/deficiency. We randomly assigned eligible patients in a 1:1 ratio to 40,000 units/week of ergocalciferol plus identical placebo or 40,000 units/week of ergocalciferol plus calcitriol (0.5 μg, two times per week) for 12 weeks.

Patients were examined at baseline, 6-week, and 12-week. Blood pressures, adverse events, concomitant drug treatment, and adherence to drug regimens were also recorded. At baseline, 6-week, and 12-week, the blood chemistry parameters including serum creatinine, cystatin C, calcium, phosphate, albumin, intact parathyroid hormone (iPTH) were measured while urine specimen collection at first void urine including urine protein to creatinine ratio (g/g) were determined. The dosages of ACEIs or ARBs were not allowed to adjust during the follow-up period. Other antihypertensive treatments were initiated or increased in dose for controlling blood pressure following the KDIGO guideline (<130/80 mmHg) [17]^.^

### Randomization and masking

Computer-generated concealed randomization schedules, each with permuted block sizes of four, were created. When an eligible patient had been enrolled, the research assistant used sealed opaque envelopes to allocate the patient to the next sequential randomization number. This study was double blinded. Patients were not informed which group they were randomly allocated to. The laboratory personnel processing the samples also had no knowledge of each patient’s group assignment. The physicians who took care of the patients were also masked.

### Outcomes

The primary endpoint was the percentage change in urine protein to creatinine ratio (UPCR) which was calculated by using the values obtained at baseline and after 12 weeks of follow-up period. Secondary endpoints were the proportion of patients who achieved at least 30% reduction in UPCR, the change of kidney functions, blood pressure level, and blood chemistry. The safety endpoints and all adverse events were classified and confirmed by personals who were masked to treatment assignment.

### Statistical analysis

The aim of this study was to determine the effect of additional calcitriol supplement in CKD patients who were receiving ergocalciferol supplement on proteinuria. As there were no published RCTs, we calculated and found that a sample size of 35 patients per arm was needed for 80% power to detect a difference in proteinuria of −1.0 g/g [standard deviation (SD) 1.5] between ergocalciferol alone and ergocalciferol plus calcitriol group with two-sided significance level of 0.05. The expected reduction in a urine proteinuria was based on our own clinical setting. The per protocol analysis was used for all efficacies and safety analyses. Patients who lost follow up and had some missing data for calculating the change values in the efficacy and safety measures between two time points were excluded from the analyses. Continuous variables were reported as mean ± standard deviation. Categorical variables were reported in terms of frequency and percentage. For normally distributed variable, the differences in changes between the two groups were analyzed by unpaired *t*-test. The differences in changes within groups were analyzed by pair *t*-test. For non-normally distributed variable, Mann–Whitney *U*-test was used to analyze differences between the two groups. Categorical outcomes were analyzed by chi-square or Fisher exact test. The statistical analyses were performed by using the SPSS version 16.0 statistical software program.

## Results

**Fig. 1:**
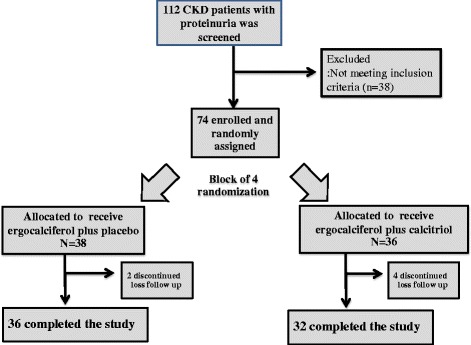
Flow diagram showing recruitment and follow-up of patients

Of 112 eligible patients, 74 were enrolled and randomly assigned to ergocalciferol plus calcitriol (combined group, *n* = 36) or ergocalciferol plus placebo (ergocalciferol group, *n* = 38). All randomly assigned patients received their allocated treatment and had at least one post-randomization follow-up visit. Two patients were lost to follow-up in ergocalciferol group and 4 patients in combined group (Fig. 1).

Baseline demographic, clinical and biochemical characteristics, and concomitant treatments were balanced between both groups. The main cause of CKD was DN (57.4%). The participants randomly allocated to the ergocalciferol group were more likely to have lower serum 25 (OH) D levels than those in the combined group (Table 1).

**Table 1:** Baseline characteristics of the study population

Demographic	Ergocalciferol plus placebo (*n* = 36)	Ergocalciferol plus calcitriol (*n* = 32)	*P*-value
Age (years)	62.56 ± 12.91	63.13 ± 11.29	0.85
Male sex (%)	15 (41.67)	17 (53.13)	0.35
Cause of CDK			
− Diabetic nephropathy	19 (52.78)	20 (62.5)	0.43
− Chronic glomerulonephritis	4 (11.11)	4 (12.5)	0.88
− IgA nephropathy	1 (2.78)	2 (6.25)	0.48
− Focal segmental glomerulosclerosis	3 (8.33)	1 (3.13)	0.35
− Polycystic kidney disease	1 (11.11)	0 (0)	0.31
− Unknown	8 (22.22)	5 (15.63)	0.49
Blood pressure (mmHg)			
− Systolic	136.69 ± 14.56	137.75 ± 14.78	0.77
− Diastolic	75.75 ± 10.46	77.13 ± 11.68	0.61
Serum creatinine (mg/dL)	2.19 ± 0.9	2.43 ± 0.9	0.29
eGFR (ml/min/1.73 m^2^)	39.29 ± 11.26	37.29 ± 11.44	0.47
Serum cystatin C (mg/L)	2.07 ± 0.61	2.13 ± 0.67	0.70
25-hydroxy vitamin D level (ng/ml)	15.89 ± 6.59	19.26 ± 5.12	0.02
Urine protein to creatinine ratio (g/g)			
− mean ± SD	3.47 ± 3.01	3.61 ± 3.75	0.87
Serum calcium (mg/dl)	9.4 ± 0.49	9.27 ± 0.47	0.27
Serum phosphorus (mg/dl)	3.58 ± 0.58	3.8 ± 0.78	0.17
Serum albumin (g/dl)	3.83 ± 0.42	3.95 ± 0.34	0.21
Serum intact parathyroid hormone (pg/mL)	85.11 ± 47.42	88.57 ± 64.49	0.60
Serum HbA1C (%)	7.17 ± 0.78	7.40 ± 0.59	0.31
Antihypertensive drug (%)			
− ARB	8 (22.22)	11 (34.38)	0.27
− ACE inhibitor	10 (27.78)	8 (25)	0.79

### Effect on proteinuria

**Fig. 2:**
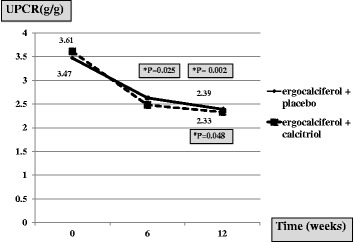
The absolute net change of UPCR from baseline to 12 weeks follow-up between both groups *P-value when compared with baseline in ergocalciferol group ^#^ *P*-value when compared with baseline in combined group

The mean baseline values of UPCR of both groups were comparable (3.6 ± 3.8 g/g in combined group and 3.5 ± 3.0 g/g in ergocalciferol group). After 12-week treatment, there were significant reductions of UPCR in both groups (2.3 ± 2.1 g/g in combined group and 2.4 ± 2.0 g/g in ergocalciferol group) (Fig. 2). The percentage reductions in UPCR of both groups were not significantly different (−25.5%, 95% CI −9.2 to −41.8 in combined group, −23.7%; 95% CI −7.0 to −40.5 in ergocalciferol group). More than 50% of patients had 30% reduction on proteinuria in both groups (56.3% in combined group and 52.8% in ergocalciferol group).

**Fig. 3:**
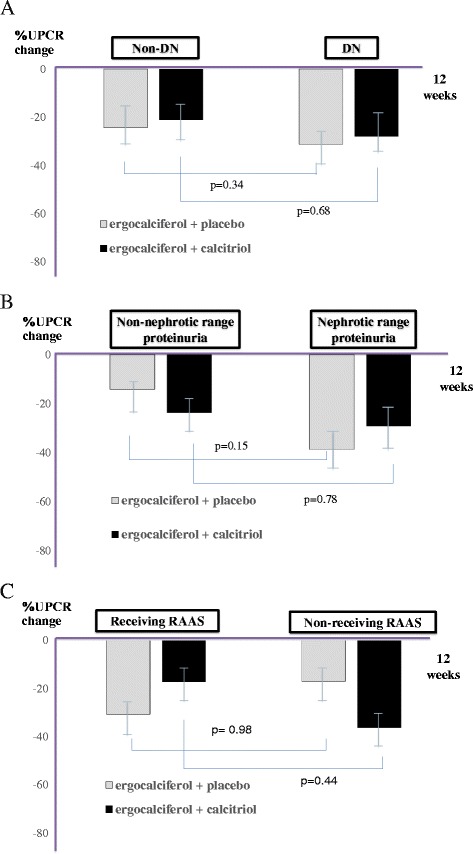
The percentage change of UPCR from baseline to 12 weeks follow-up between both groups. Subgroup analyses by cause of CKD (3A), Level of proteinuria (3B), Receiving RAAS (3C)

Subgroup analysis by cause of CKD, the level of proteinuria, and status of receiving RAAS blockade were performed. The mean change in UPCR from baseline to the end of treatment in DN seemed to be greater than non-DN [−31.3% vs. -15.3% in ergocalciferol group; and −28.1% vs −21.2% in combined group] (Fig. 3a). In addition, the mean change in UPCR seemed to be more robust in case of nephrotic range proteinuria than non-nephrotic range [−38.7% vs −14.2% in ergocalciferol group; and −28.7% vs −23.8% in combined group (Fig. 3b). However, the mean changes in UPCR in whom receiving RAAS blockade or non-receiving RAAS blockade were not significantly different. [−30.6% vs −16.9% in ergocalciferol group; and −17.2% vs −36.2% in combined group] (Fig. 3c).

### Effect on kidney function

**Fig. 4:**
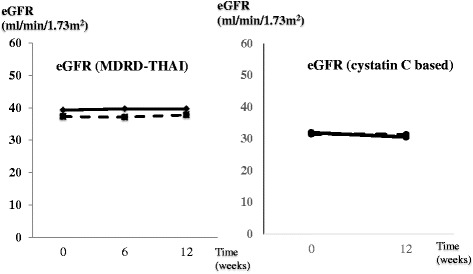
The absolute net change of eGFR (MDRD-THAI) and eGFR (CKD-epi cystatin c based) from baseline to 12 weeks follow-up between both groups

eGFR determined by serum creatinine (Thai MDRD) and by cystatin C (CKD-EPI) did not change during the study period from baseline to the end of treatment and there were no significant differences between the ergocalciferol group and the combined group (Fig. 4).

### Effect on blood pressure

There were no significant changes in mean systolic and diastolic blood pressure values throughout the course of study in either ergocalciferol group or combined group. Mean blood pressure values at baseline were 136.7 ± 14.6/75.8 ± 10.5 mmHg in the ergocalciferol group and 137.8 ± 14.8/77.1 ± 11.7 mmHg in the combined group (NS). At the end of treatment, mean blood pressure values were 135.6 ± 17.4/77.9 ± 11.2 mmHg in the ergocalciferol group and 135.8 ± 20.0/77.9 ± 11.9 mmHg in the combined group.

### Effect on metabolic bone disease

There were no significant changes of serum calcium, serum phosphate from baseline to the end of treatment as well as between both groups [9.4 ± 0.5 to 9.3 ± 0.6 mg/dL and 3.6 ± 0.6 to 3.8 ± 0.7 mg/dL in ergocalciferol group, 9.3 ± 0.5 to 9.2 ± 0.5 mg/dL and 3.8 ± 0.8 to 3.8 ± 0.6 mg/dL in combined group]. No severe hypercalcemia, hyperphosphatemia, or serious side effects were noted in both groups (NS).

There were no significant differences between iPTH levels measured at baseline between two groups. Serum iPTH was slightly increased from 85.1 ± 47.4 pg/mL at baseline to 86.0 ± 46.0 pg/mL (*P* = 0.83) at the end of treatment in the ergocalciferol group and was significantly decreased from 88.6 ± 64.5 to 73.9 ± 73.3 pg/mL (*P* = 0.04) in the combined group. Serum iPTH levels in the combined group were significantly lower than the ergocalciferol group at the end of treatment (*P* = 0.03).

Vitamin D levels (25(OH)D) were significantly increased from baseline to the end of treatment in both ergocalciferol group (15.9 ± 6.6 to 30.6 ± 11.9 ng/mL) and combined group (19.5 ± 5.2 to 33.4 ± 11.3 ng/mL), but no statistical significance was observed between both groups.

## Discussion

This study was the first double-blind RCT to explore the additional benefit of calcitriol supplement in CKD patients who were receiving ergocalciferol supplementation on proteinuria and kidney function by using serum cystatin C in CKD patients with vitamin D insufficiency/deficiency. The study demonstrated that the 12-week course of ergocalciferol supplementation in patients with stage 3–4 CKD and nutritional vitamin D insufficiency/deficiency could improve proteinuria as well as restored 25-vitamin D concentrations. Nevertheless, adding calcitriol to ergocalciferal treatment did not provide further beneficial effect for proteinuria (Fig. 2). Moreover, the kidney function monitored by serum creatinine-based and cystatin C-based eGFR did not significantly change from the baseline to the end of treatment in both groups and there were no significant differences between the two groups (Fig. 4). No severe hypercalcemia or serious side effects were noted in both ergocalciferol and combined groups. In addition, blood pressure and glycemic control were not significantly different between two groups.

Although several observational studies in CKD patients have shown the benefit of nutritional vitamin D supplementation in decreasing proteinuria [10, 11], there were no RCTs exploring the benefit of nutritional vitamin D on proteinuria and renal functions. This RCT demonstrates the benefit of nutritional vitamin D supplement in proteinuric CKD with vitamin D insufficiency/deficiency on proteinuria. The potential mechanisms remain to be further explored. One possible mechanism is that normal ranges of 25-(OH) D level achieved by ergocalciferol supplement might be changed to the active form and could activate VDR.

Several previous RCTs of proteinuric kidney disease demonstrated the beneficial effect of calcitriol or paricalcitol supplement on decreasing proteinuria [13–15]. However, there is some inconsistency of the evidence. In RCT of selective vitamin D receptor activation with paricalcitol for reduction of albuminuria in patients with type 2 diabetes (VITAL study), paricalcitol could not demonstrate an additional effect in decreasing albuminuria in DN patients [18]. The different dosage regimens and the levels of proteinuria might affect the results. Indeed, in a recent systematic review and meta-analysis, active vitamin D, vitamin D compounds, and nutritional vitamin D showed antiproteinuric effect in CKD patients [19, 20]. However, these are no available data regarding the role of combined nutritional vitamin D and active vitamin D on proteinuria and renal function in CKD patients with vitamin D insufficiency/deficiency. In the present study, the combined ergocalciferol and calcitriol also resulted in decreasing proteinuria from baseline while the additional effect of combined nutritional and active vitamin D could not be demonstrated (Fig. 2). Admittedly, the long-term benefit of combined ergocalciferol and calcitriol was not explored in this study.

As such, the present study demonstrates that whenever nutritional vitamin D is supplemented until achieving the target level, the agent can decrease proteinuria. Vitamin D and its analogues appear to have similar antiproteinuric effects through activation of the VDR by several mechanisms. Active vitamin D acts as a strong negative endocrine regulator of the RAAS and functions mainly to suppress renin production in experimental studies [21, 22]. Inactive vitamin D and its metabolites (such as 25(OH) D and 24,25 (OH)2 D) might also have additional effect on proximal tubular cells. 25(OH) D bound to vitamin D binding protein is endocytosed by megalin-cubilin in the apical membrane of proximal tubular cells. Then, intracellular 25(OH) D is metabolized to 1, 25(OH) 2D (calcitriol) by 1-α-hydroxylase in the tubular cell [23]. Furthermore, vitamin D has been shown to have anti-fibrotic and immunomodulatory effects by attenuating fibrotic and inflammatory markers in animal and clinical studies [24–26]. The potential mechanism should be explored in further studies.

In term of kidney function, vitamin D compounds could not demonstrate the benefit on renal function in CKD patients in a recent systematic review and meta-analysis [19, 20]. However, the renal function in all included studies was determined by serum creatinine-based formula which might be interfered by increasing muscle mass from vitamin D supplement. New biomarkers such as serum cystatin C should be used for more accurate renal function measurement. Although the serum cystatin C was used for calculating eGFR in this study (Fig. 4), the benefits of kidney function after vitamin D supplement were still not proven. A longer study might be required to examine the renal function retardation effect of ergocalciferol with/without calcitriol treatment.

Regarding metabolic bone disease, although serum calcium and phosphate levels did not change during the treatment in both groups, the benefit of controlling serum iPTH was demonstrated only in the calcitriol group. Therefore, active vitamin D compound should be used in CKD if the goal is to treat secondary hyperparathyroidism. There are some limitations of the present analysis including small sample size that might result from unexpected high standard deviation, the method of urine protein measurement, office blood pressure monitoring, and short-term follow up.

Finally, according to the 2003 KDOQI guideline, it remains controversial regarding the definite critical serum 25 (OH) D level and benefits of 25 (OH) D supplements in CKD patients [3]. The guideline only mentioned that whenever serum 25(OH) D levels < 30 ng/mL, supplementation with nutritional vitamin D should be initiated using dosing regimens recommended for the general population. The first line use of ergocalciferol or cholecalciferol was recommended due to less hypercalcemia and hyperphosphatemia [4]. In another guideline, the Kidney Disease: Improving Global Outcomes (KDIGO) guideline [27] also provided opinion-based recommendations regarding measurement of 25(OH) D in patients with CKD. KDIGO recommended correcting 25(OH) D deficiency or insufficiency using treatment strategies used for the general population. Although ergocalciferol-prescribing strategy in hemodialysis patients with vitamin D insufficiency/deficiency using the KDOQI and KDIGO guidelines is inadequate to achieve repletion or maintenance of normal vitamin D levels [28], the ergocalciferol dosing in this study can improve and maintain vitamin D status in CKD patients.

## Conclusions

The effect of ergocalciferol to lower proteinuria in CKD patients with vitamin D deficiency was demonstrated in this RCT. However, additional calcitriol did not have more proteinuria-lowering effects. Therefore, ergocalciferol should be added for more decreasing proteinuria in CKD patients with proteinuria that cannot tolerate or increase the dosage of RAAS blockade due to any side effects. However, there are some limitations of the present analysis including small sample size and short-term follow up that might result in non-significance on renal function.

## Abbreviations

25-OH-D: 25-hydroxy vitamin D
ACEI: Angiotensin-converting-enzyme inhibitor
ARBs: Angiotensin II receptor blockers
CKD: Chronic kidney disease
CKD-EPI: The Chronic Kidney Disease Epidemiology Collaboration
DN: Diabetic nephropathy
eGFR: Estimated glomerular filtration rate
ESRD: End-stage renal disease
iPTH: Intact parathyroid hormone
KCMH: King Chulalongkorn Memorial Hospital
KDIGO: Kidney disease improving global outcomes
KDOQI: Kidney Disease Outcomes Quality Initiative
MBD: Mineral and bone disorder
MDRD: The Modification of Diet in Renal Disease
RAAS: Renin angiotensin aldosterone system
RCT: Randomized controlled trial
SD: Standard deviation
TCTR: Thai Clinical Trials Registry
UPCR: Urine protein to creatinine ratio
VDR: Vitamin D receptor
